# A Prolonged Outbreak of Enteric Fever Associated With Illegal Miners in the City of Matlosana, South Africa, November 2020–September 2022

**DOI:** 10.1093/ofid/ofae118

**Published:** 2024-02-28

**Authors:** Phuti Sekwadi, Anthony Marius Smith, Wellington Maruma, Gift Mongologa, Grace Tsele, Mimmy Ngomane, Nomsa Tau, Shannon Williams, Bolele Disenyeng, Mahlaku Sebiloane, Leigh Johnston, Linda Erasmus, Juno Thomas

**Affiliations:** Centre for Enteric Diseases, National Institute for Communicable Diseases, National Health Laboratory Service, Johannesburg, South Africa; Centre for Enteric Diseases, National Institute for Communicable Diseases, National Health Laboratory Service, Johannesburg, South Africa; Department of Medical Microbiology, Faculty of Health Sciences, School of Medicine, University of Pretoria, Pretoria, South Africa; Division of Public Health Surveillance and Response, National Institute for Communicable Diseases, National Health Laboratory Service, Johannesburg, South Africa; Julius Global Health, Julius Center for Health Sciences and Primary Care, University Medical Centre Utrecht, Utrecht University, Utrecht, the Netherlands; Health Programmes Directorate, North West Provincial Department of Health, Mahikeng, South Africa; Health Programmes Directorate, North West Provincial Department of Health, Mahikeng, South Africa; Centre for Enteric Diseases, National Institute for Communicable Diseases, National Health Laboratory Service, Johannesburg, South Africa; Centre for Enteric Diseases, National Institute for Communicable Diseases, National Health Laboratory Service, Johannesburg, South Africa; Centre for Enteric Diseases, National Institute for Communicable Diseases, National Health Laboratory Service, Johannesburg, South Africa; Centre for Enteric Diseases, National Institute for Communicable Diseases, National Health Laboratory Service, Johannesburg, South Africa; South African Field Epidemiology Training Program, National Institute for Communicable Diseases, National Health Laboratory Service, Johannesburg, South Africa; South African Field Epidemiology Training Program, National Institute for Communicable Diseases, National Health Laboratory Service, Johannesburg, South Africa; Centre for Enteric Diseases, National Institute for Communicable Diseases, National Health Laboratory Service, Johannesburg, South Africa; Centre for Enteric Diseases, National Institute for Communicable Diseases, National Health Laboratory Service, Johannesburg, South Africa

**Keywords:** carrier, enteric fever, illegal miner, prolonged outbreak

## Abstract

**Background:**

In South Africa, the annual incidence of enteric fever averaged 0.1 per 100 000 persons between 2003 and 2018. During 2021 an increase in the number of enteric fever cases was observed. An outbreak investigation was conducted to determine the magnitude and source of the outbreak.

**Methods:**

We performed a cross-sectional descriptive study. Data were collected through telephonic or face-to-face interviews with cases or proxies via a standardized case investigation form. Whole genome sequencing was performed on all *Salmonella* Typhi isolates. Drinking water samples were collected, tested, and analyzed. Descriptive analysis was performed with Microsoft Excel.

**Results:**

Between January 2020 and September 2022, a cluster of 53 genetically highly related *Salmonella* Typhi isolates was identified from 5 provinces in South Africa. Isolates associated with the cluster showed ≤5 allelic differences, as determined following core genome multilocus sequence typing analysis. Most cases (60%, 32/53) were in the North West province. Males represented 68% (36/53). Of these, 72% (26/36) were aged 15 to 49 years, with a median age of 31 years. Where occupation was known within this age group, 78% (14/18) were illegal gold miners. Illegal miners reported illness onset while working underground. Five municipal tap water samples were tested and showed no evidence of fecal contamination.

**Conclusions:**

This outbreak predominantly affected illegal gold miners, likely due to the consumption of contaminated groundwater while working in a gold mine shaft. In addition, this investigation highlights the value of whole genome sequencing to detect clusters and support epidemiologic investigation of enteric fever outbreaks.

## Teaser

An increase in the number of enteric fever cases was observed in South Africa between 2020 and 2022. The outbreak predominantly affected illegal gold miners, likely due to the consumption of contaminated water while underground.

Enteric fever is a potentially life-threatening bacterial infection caused by *Salmonella enterica* serovar Typhi or Paratyphi A, B, or C. Enteric fever collectively refers to typhoid fever caused by *S enterica* serovar Typhi (*Salmonella* Typhi) and paratyphoid fever caused by *S enterica* serovar Paratyphi [1]. The disease is transmitted through consumption of food or water contaminated by the bacteria, while the presence of chronic carriers results in a sustained presence of new cases in communities [2–4]. Without treatment approximately 2% to 5% of cases will become chronic carriers [5]. Prolonged fever is the cardinal symptom of enteric fever, and other symptoms may include fatigue, abdominal pain, diarrhea, or constipation [5, 6]. Severe disease may be complicated by intestinal perforation, gastrointestinal bleeding, or encephalopathy and may lead to death [7].

According to the World Health Organization, an estimated 11 to 20 million cases of enteric fever resulting in 128 000 to 161 000 deaths occur every year worldwide [5]. The disease is common in low- to middle-income countries and is often associated with poor access to clean water and inadequate sanitation. South Asia, Southeast Asia, and sub-Saharan Africa bear the highest burden of enteric fever disease [8–11]. A study on the burden of enteric fever diseases conducted in Bangladesh, Nepal, and Pakistan reported incidence rates of 913, 330, and 176 cases per 100 000 population, respectively [9]. South Africa is endemic for enteric fever caused by *Salmonella* Typhi, although the prevalence of disease is much lower than most other countries in sub-Saharan Africa, with an average annual incidence of 0.1 per 100 000 population between 2003 and 2018. Enteric fever caused by *Salmonella* Paratyphi remains rare in South Africa [12]. Enteric fever is a notifiable medical condition in South Africa; however, cases significantly underrepresent the true burden of disease.

**Figure 1.**
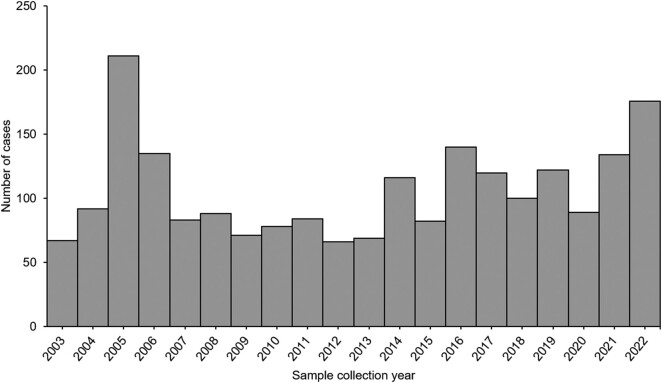
Cases of laboratory-confirmed enteric fever, South Africa, 1 January 2003–22 September 2022.

Following the last large outbreak of typhoid fever in Delmas, Mpumalanga province, in 2005 [13], with >1000 probable and laboratory-confirmed cases, the number of enteric fever cases in South Africa has declined over the last few decades, and larger outbreaks have become less common. From 2006 through 2021, the number of laboratory-confirmed cases of enteric fever averaged 99 per year (range, 66–140). Between 1 January and 22 September 2022, 176 cases of enteric fever were reported, the highest number within a year since the outbreak in Delmas (Figure 1).

On 24 November 2021, the Centre for Enteric Diseases (CED) of the National Institute for Communicable Diseases (NICD) was notified of an increase in the number of enteric fever cases diagnosed at a hospital complex (hospital A) within the City of Matlosana in the North West province. A review of laboratory surveillance data for 2020 and 2021 confirmed an increase of laboratory-confirmed enteric fever diagnoses at hospital A during 2021. Analysis of the *Salmonella* Typhi isolates by whole genome sequencing and core genome multilocus sequence typing (cgMLST) showed that >90% (16/17) of the initial isolates were part of a highly genetically related cluster or outbreak. Outbreak investigation teams at various levels were constituted and activated to determine the magnitude and source of the outbreak and to recommend relevant control and prevention measures.

## METHODS

### Study Setting

The outbreak predominantly affected people living within the City of Matlosana local municipality: the smallest but most populated of the 3 local municipalities within the Dr Kenneth Kaunda District in terms of area. The city is known for its gold mines that were established in the 1900s. Some of the mining shafts have been closed, and a few shafts remain in operation [14]. The municipality comprises several communities located around the city, with a total population of about 398 676 as reported in the 2011 national census. Formal housing accounts for 83% of households, and 93% of all households have access to flushing toilets and treated municipal running water [15].

### Study Design

We performed a descriptive cross-sectional study. In-depth phone or in-person interviews were done with cases who had been diagnosed with laboratory-confirmed enteric fever brought on by a genetically related strain of *Salmonella* Typhi between January 2020 and September 2022. Proxies were interviewed when cases were not available.

### Epidemiologic Investigations

An outbreak investigation team visited the affected area to investigate in November and December 2021. Telephonic and face-to-face interviews were ongoing for cases diagnosed during 2022. Trend analysis was performed on historical enteric fever laboratory data.

### Laboratory Investigations

The surveillance of enteric fever in South Africa is laboratory based. All laboratory-confirmed cases diagnosed at public and private laboratories are reported to the department of health and the NICD-CED through the Notifiable Medical Conditions System. Blood culture is the primary method for enteric fever diagnosis in private and public laboratories in South Africa. All isolates of *Salmonella* Typhi are sent to NICD-CED for confirmation and further characterization.

### Laboratory Investigation Methodologies

The CED received *Salmonella* Typhi on Dorset-Egg transport media (Diagnostic Media Products, National Health Laboratory Service) and subcultured onto 5% blood agar (Diagnostic Media Products) to check for viability and purity. Cultures were identified by standard phenotypic microbiological identification and serotyping techniques, briefly described as follows. As required, bacterial colonies were identified with the VITEK-2 COMPACT 15 automated microbial identification system (bioMérieux). Serotyping was performed according to the White-Kauffmann-Le Minor scheme [16]. Antimicrobial susceptibility testing was performed via the Etest method (bioMérieux). Interpretation of antimicrobial susceptibility data was done in accordance with the Clinical and Laboratory Standards Institute [17].

Genomic DNA was isolated from bacteria with an Invitrogen PureLink Microbiome DNA Purification Kit. Whole genome sequencing was performed by Illumina NextSeq next-generation sequencing technology, with DNA libraries prepared by a Nextera DNA Flex Library Preparation Kit (Illumina), followed by 2 × 150–base pair paired-end sequencing runs with ∼80-times coverage. Illumina paired-end reads were analyzed by the JEKESA bioinformatics pipeline (https://github.com/stanikae/jekesa), briefly described as follows. Quality control and read filtering of raw reads were performed with FastQC and TrimGalore. Species identification and closest reference detection were performed with BactInspector. Contamination checks were performed by ConFindr and Kraken. Raw reads were assembled by using SKESA, SPAdes, MEGAHIT, or Velvet as implemented in Shovill. The final assembly was assessed with QUAST. The assembled genomes were further investigated with the following tools. Multilocus sequence typing was performed by using tools at the PubMLST platform (https://pubmlst.org/). Detection of antimicrobial resistance determinants was performed with ResFinder and PointFinder (http://www.genomicepidemiology.org/services/). *Salmonella* serovar prediction was performed with SeqSero2 (http://denglab.info/SeqSero2) and SISTR [18]. Plasmid DNA presence was investigated with PlasmidFinder (http://www.genomicepidemiology.org/services/). Information regarding *Salmonella* Typhi genotype, as called by single-nucleotide polymorphism analysis, was determined by the GenoTyphi scheme [19] at the Pathogenwatch platform (https://pathogen.watch/). cgMLST was used to investigate the phylogeny and genetic relatedness of isolates. This was made possible by using the cgMLST scheme available at the EnteroBase platform (http://enterobase.warwick.ac.uk/species/index/senterica). Raw sequencing data (FastQ files for paired-end reads) were uploaded and investigated at EnteroBase via the cgMLST tool (with the cgMLST V2 + HierCC V1 scheme, which incorporates analysis at 3002 genes). The phylogeny and genetic relatedness of isolates was depicted by a GrapeTree-generated minimum spanning tree per the MSTree V2 algorithm. A cluster of isolates or outbreak was defined as ≥3 isolates showing ≤5 allelic differences, as determined following cgMLST analysis and visualized on a GrapeTree-generated minimum spanning tree.

All sequencing data were uploaded to the public EnteroBase platform (http://enterobase.warwick.ac.uk/species/index/senterica) and so are freely available to access. In addition, all sequencing data were deposited in the European Nucleotide Archive (project accession PRJEB39988).

Rectal swabs collected from the contacts of the cases during the outbreak investigation were cultured for *Salmonella* Typhi at the NICD-CED laboratory.

### Environmental Investigations

Water samples were collected from running water taps within the houses of the confirmed cases within the City of Matlosana and tested for *Salmonella* Typhi and total coliform count. The routine municipal water quality indicator test results from the affected areas were reviewed.

### Data Collection and Analysis

Enteric fever laboratory surveillance data were obtained from the NICD-CED. Patient demographics, disease presentation, and exposure details were collected through the completion of a standardized NICD form for enteric fever case investigation. Additional data, including cases’ residential addresses and contact details, were obtained from the hospitals at which the cases were admitted. Data are kept on a password-encrypted Microsoft Excel document. Data cleaning and descriptive analyses were completed with Microsoft Excel.

## RESULTS

### Epidemiologic Findings

**Figure 2.**
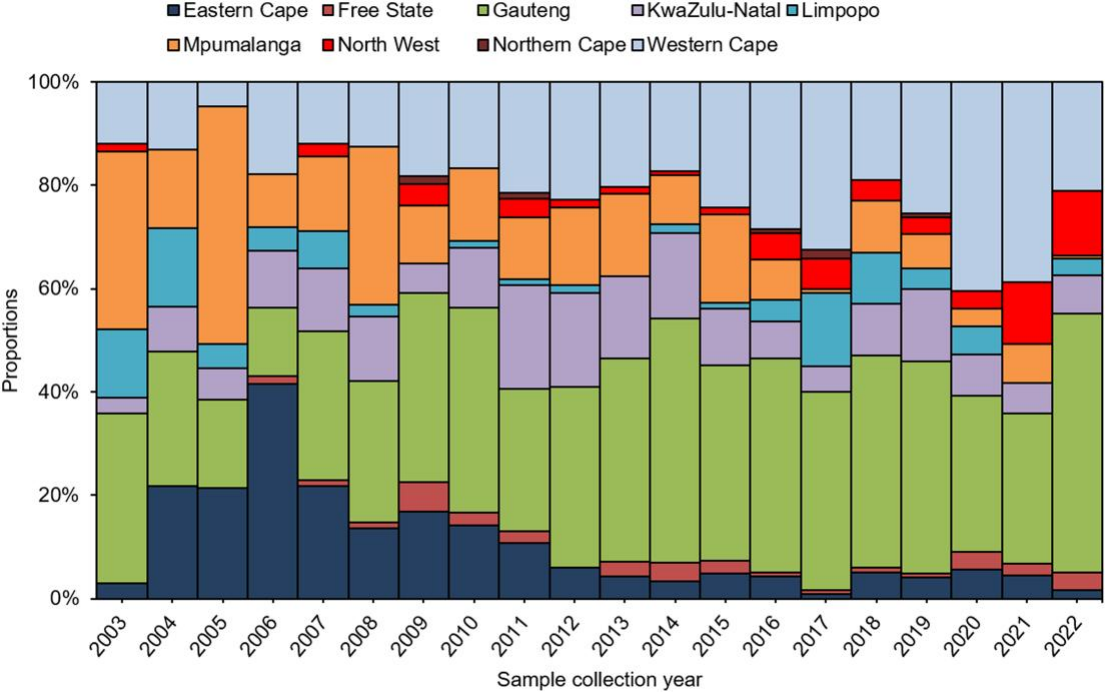
Annual proportions of laboratory-confirmed cases of enteric fever by province, South Africa, 1 January 2003–22 September 2022.

**Figure 3.**
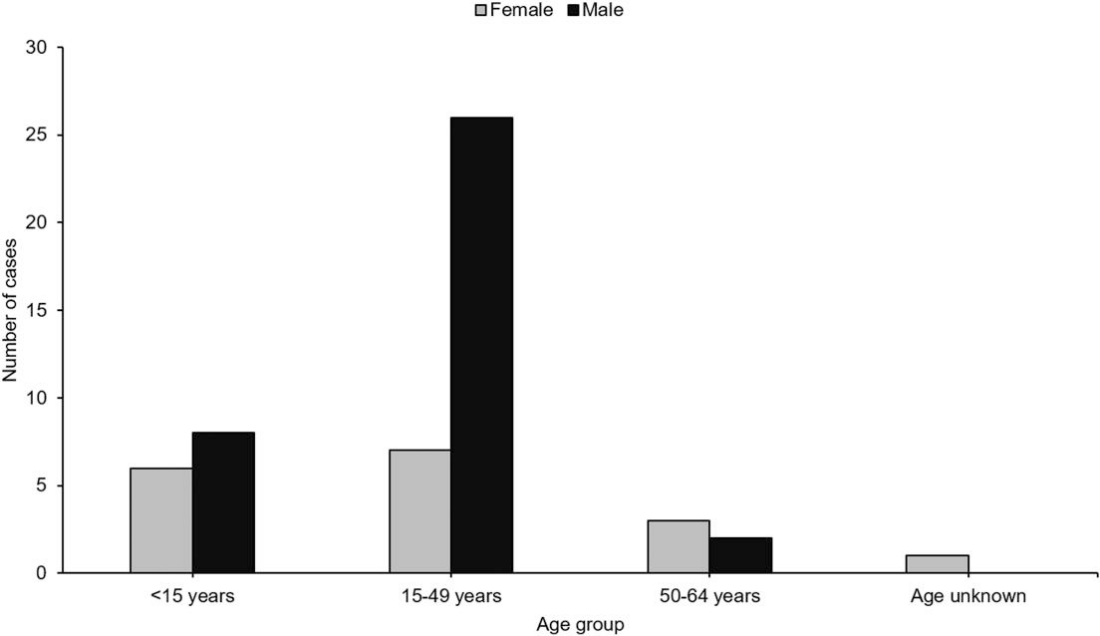
Distribution of laboratory-confirmed outbreak cases of enteric fever by sex and age, South Africa, 1 January 2003–22 September 2022 (N = 53).

Between January 2020 and September 2022, 53 cases of enteric fever of the outbreak strain were reported from 5 provinces in South Africa. During the same period, 346 nonoutbreak-related cases were reported. The majority of the outbreak cases were from the North West province (60%, 32/53), followed by Gauteng (25%, 13/53) and Mpumalanga (8%, 4/53); Free State and KwaZulu-Natal had 2 cases each (4%, 2/53). By comparison, the North West had an annual average of 2 cases between 2003 and 2019 (Figure 2). Case investigation forms were completed for 57% (30/53) of cases, and occupation was recorded for 68% (26/38) of cases aged ≥15 years. The age group of 15 to 49 years accounted for most cases (62%, 33/53), followed by children aged <16 years (26%, 14/53) and persons aged 50 to 64 years (9%, 5/53). Males constituted 68% (36/53) of cases. Of these, 72% (26/36) were within the age group of 15 to 49 years, with a median age of 31 years (range, 15–43; Figure 3).

All cases reported fever; other symptoms were fatigue (83%, 25/30), diarrhea (63%, 19/30), abdominal pain (50%, 15/30), vomiting (43%, 13/30), and constipation (17%, 5/30). Three deaths were noted (2 male adults and a female child). Of 14 cases with known HIV status, 2 (14%) were HIV infected. Where illness onset was known (55%, 29/53), the median time between illness onset and hospital admission was 2 weeks (range, 0–8). Of the 30 cases interviewed, 9 developed complications, which included renal failure (3/9), hepatomegaly (2/9), intestinal perforation, encephalopathy, and miscarriage.

The index case was an adult female with a travel history to Mozambique. She sought medical care in the North West Province but reported onset of illness while in Mozambique. Her household contacts included her father and sister, who were subsequently diagnosed with enteric fever. Her partner, who shared accommodation with her, worked as an illegal gold miner, although he remained asymptomatic.

**Figure 4.**
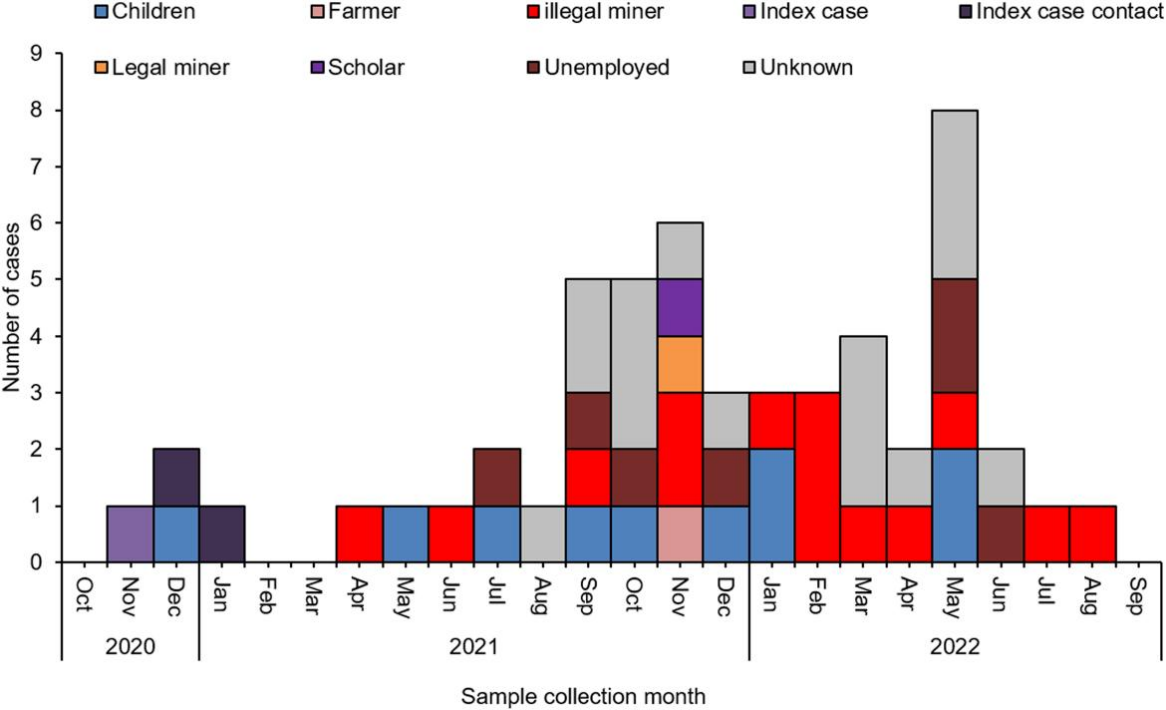
Epidemic curve of laboratory-confirmed outbreak cases of enteric fever by occupation, South Africa, 1 January 2003–22 September 2022 (N = 53).

Of the 26 males within the age group of 15 to 49 years, occupation was known for 69% (18/26), of which 78% (14/18) were illegal gold miners (also known as *Zama Zamas*; in South Africa, this is an isiZulu phrase meaning “keep on trying”), working at a specific abandoned gold mine shaft in the City of Matlosana (Figure 4). All Zama Zamas reported illness onset while working underground. They reported living and working underground (without surfacing) for an average period of 5 months (range, 1–11). The mining shafts were dark, poorly ventilated, and overcrowded with approximately 300 miners on each level at any time, with no toilets or running water. Groundwater flowing from the upper aquifers flooded underground workings, and the same water was used for drinking. Bodies of stagnant water were sometimes created within the abandoned shafts. While underground, the Zama Zamas defecated in the same areas where they accessed drinking water. Accumulation of groundwater in the mining shafts in the City of Matlosana goldfields has been reported in literature [14, 20].

One of the 3 Zama Zamas diagnosed in Gauteng province and a Zama Zama from Free State province reported falling ill while working at the City of Matlosana gold mine shaft. The second case from the Free State province was a child. Her proxy stated that, prior to the child's onset of illness, her uncle had displayed similar symptoms and reported illness onset while working as a Zama Zama at the same mine in the City of Matlosana. The other 2 Zama Zama cases from Gauteng province did not disclose the location of the mines where they worked.

Eight interviewed cases reported no contact with Zama Zamas or working in the mines. Where data were available, most cases (82%, 19/22) reported living in a formal house with access to treated municipal running tap water and flushing toilets. No other epidemiologic links were identified among the cases, besides the Zama Zamas who worked in the same shaft before illness onset. No common food items sourced from common food places were reported by the cases.

### Laboratory Findings

**Figure 5.**
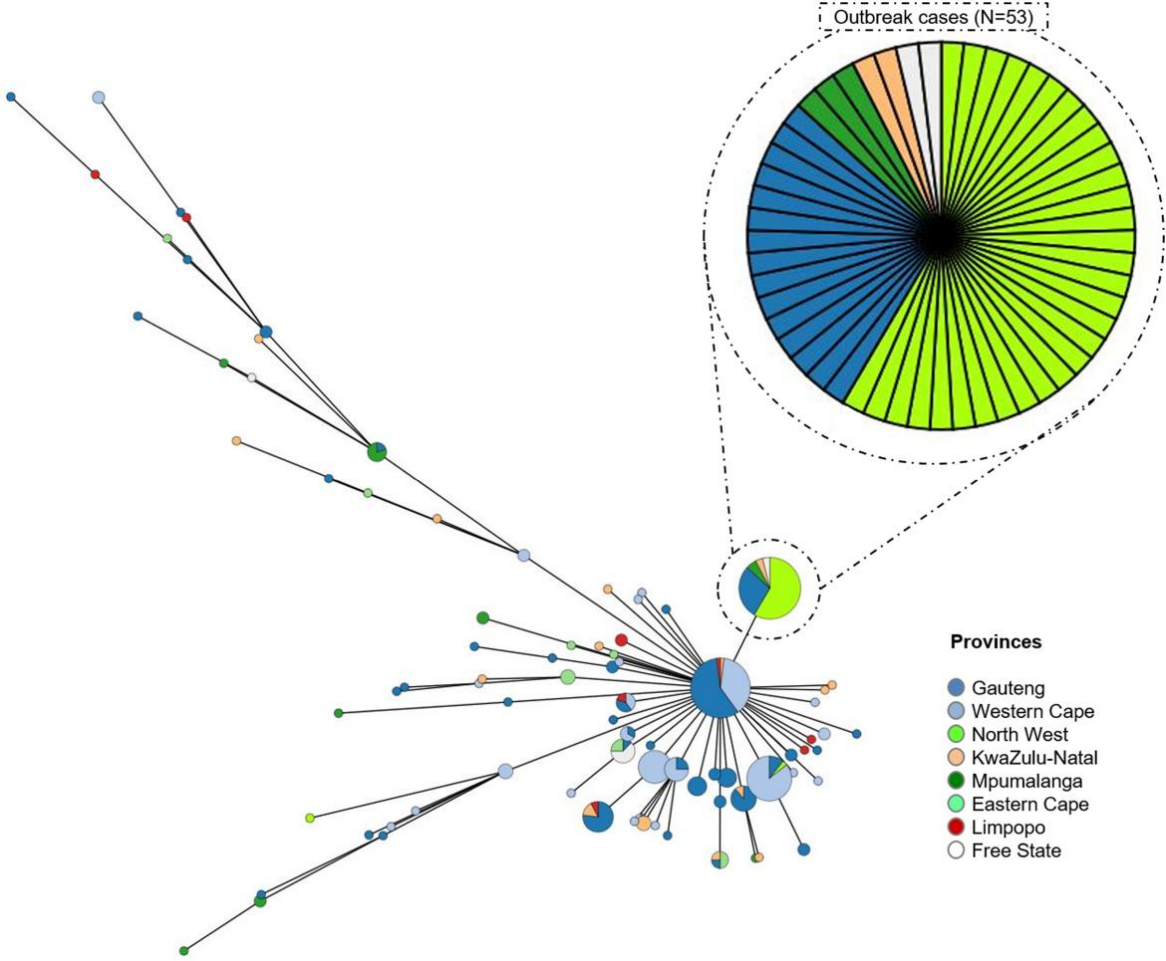
Minimum spanning tree drawn according to cgMLST data from *Salmonell*a Typhi isolates sourced from South Africa, 2020–2022. The circular nodes represent isolates. Isolates showing ≤5 allelic differences are collapsed into a single circular node. cgMLST, core genome multilocus sequence typing.

All cases were diagnosed by isolation of *Salmonella* Typhi from blood culture. All 53 *Salmonella* Typhi isolates associated with the outbreak showed ≤5 allelic differences, as determined following cgMLST analysis and visualized on a GrapeTree-generated minimum spanning tree (Figure 5), which is indicative of genetically highly related isolates. All isolates were multilocus sequence type 1; all showed a GenoTyphi genotype 4.3.1.1.EA1; and all showed a multidrug-resistance genotype with the presence of multiple antimicrobial-resistance genes: *bla*_TEM-1B_, *sul1*, *sul2*, *catA1*, *dfrA7*, *aac(6)-Iaa*, *aph(6)-Id*, and *aph(3'')-Ib*.

Phenotypically, all 53 isolates were resistant to ampicillin but susceptible to azithromycin, ceftriaxone, and ciprofloxacin. Azithromycin is the drug of choice for treatment of enteric fever in South Africa. Ciprofloxacin is the empiric treatment of choice for uncomplicated enteric fever in South Africa. When antimicrobial susceptibility results are available, the treatment can be tailored accordingly.

Eleven rectal swabs collected from household contacts of 6 cases all tested negative for *Salmonella* Typhi on culture.

### Environmental Findings

*Salmonella* Typhi was not isolated from 5 municipal tap water samples collected from affected households, and the total coliform counts were within the limit for drinking water in South Africa (<10 colony-forming units/100 mL).

## DISCUSSION

An unusual feature of this outbreak of enteric fever was that it affected predominantly males of working age rather than children aged <15 years, as is more commonly described [5, 21–23]. We hypothesized that primary exposure to the source of infection was limited to this particular group, namely Zama Zamas working in an abandoned mine shaft in the City of Matlosana. Although we could establish occupation in only 26 of 53 cases, this theory is supported by the fact that 54% reported to be Zama Zamas, and they became ill while working underground. No other common occupation was reported by ≥2 people, while the age group affected could suggest a common occupation or social activity. The illegal nature of Zamas Zamas’ work makes it likely that this occupation would be underreported. Enteric fever has a median incubation period of 2 weeks [24], and miners spend an average of 5 months underground, limiting the possible place of exposure to the mines. Other than illegal mining activities reported by the miners, there were no common activities shared among the cases, and they did not know one another. Despite living in different provinces and communities, definite epidemiologic links to the mine shaft in the City of Matlosana were established among some of the cases.

The prolonged nature of the outbreak is likely due to the presence of acutely ill persons [25] and possibly chronic carriers [26, 27] working and living in the shafts, continually shedding the bacteria in their feces and leading to ongoing contamination of the groundwater sources within the shafts. Prolonged outbreaks of enteric fever have been associated with the presence of chronic carriers [26].

It is likely that the *Salmonella* Typhi strain in this outbreak was imported from Mozambique through the index case who got ill while in Mozambique. Subsequently, the strain got introduced into the mine shafts in the City of Matlosana by the partner of the index case, who is likely an asymptomatic carrier. A study in Western Sydney identified 2 siblings who contracted enteric fever in 2013, and investigations identified the father as a chronic carrier. The father had traveled to Bangladesh in 2011 but never developed symptoms [28]. A similar outbreak was reported in Japan, where a returning traveler, also a chef, was identified as an asymptomatic carrier [29]. In our study, 3 months after the family cluster was determined, the first hospitalized Zama Zama was identified in April 2021; subsequently, on average, a Zama Zama belonging to the outbreak was reported every month between April 2021 and September 2022.

Contamination of municipal water was considered an unlikely source of the outbreak, as most of the cases cited access to safe water and adequate sanitation while on the surface; in addition, the cases reside in 5 provinces, with different sources of municipal water supplies from different reservoirs. Municipality-supplied running tap water in the City of Matlosana showed coliform counts within acceptable limits. In contrast to this slow-growing and prolonged outbreak, outbreaks of enteric fever associated with drinking municipal water are typically associated with a sudden increase in the number of cases and affect mostly vulnerable groups, including children [22, 30].

The presence of cases that had no contact with Zama Zamas or illegal mining activities suggests community transmission. When Zama Zamas resurface from the mines, they return to live with their families within their communities. Those who are acutely ill and asymptomatic carriers may continue spreading the infection to their household contacts [31] and possibly people within the community. Some of the infected Zama Zamas travel to other provinces and neighboring countries. This increases the risk of potential contamination of municipal water sources and food sources in the community, which could result in large water- or foodborne outbreaks of enteric fever.

Enteric disease outbreaks among miners have been reported in South Africa and other African countries. In 2002 an outbreak of cholera occurred at a gold mine in the North West Province affecting miners and administration staff. Twelve laboratory-confirmed cases were identified, with 9 of the cases working in the same shaft [32]. In 2010, an outbreak of cholera occurred among a group of small-scale miners in the Eastern Region of Ghana. A total of 136 cases were recorded, with 56% being males, of which 40% were either miners or coming from households of miners [33].

All cases in this outbreak were hospitalized and 3 died. Complications were noted among 9 of the 30 interviewed cases. When compared with other studies, the rate of complications was higher in this outbreak [34–36], most likely due to delayed presentation [22, 37–39]. Over 70% of Zama Zamas in South Africa are illegal immigrants [40], which may result in hesitancy to seek health care. Among those who reported illness onset, the median period before presentation to hospital was 14 days.

An additional consequence of not seeking health care is that without treatment some of the cases will progress to carrier status and continue shedding the bacteria, increasing the risk of food and water contamination within the communities and in the mines.

The outbreak team recommended several interventions: health education, community messaging around hand hygiene and food/water safety, raising awareness among health care workers, and ongoing monitoring of municipal water sources. Most important was the need to develop and implement targeted health education to inform Zama Zamas of the high risk of enteric fever and the use of preventive measures while working underground, including water treatment tablets or household bleach to treat water for drinking or cooking and the use of alcohol-based sanitizer for cleaning hands before eating. Prompt interventions can help prevent larger outbreaks and minimize the risk of a similar outbreak occurring in the future.

### Limitations

Case investigation forms could be completed for only 57% of the cases. The illegal nature of Zama Zamas’ work gave rise to a number of limitations: reluctance of cases or proxies to disclose their occupation, failure to disclose the identity of the shaft or definitively identify the location of the shaft, and inability of the outbreak team to enter the mine to collect and test water samples. Many Zama Zamas in South Africa are illegal immigrants [40] and are hesitant to seek health care, probably resulting in missed cases. A case-control study could not be conducted due to a lack of appropriate controls.

## CONCLUSION

We described an outbreak of enteric fever predominantly affecting Zama Zamas, likely due to the consumption of contaminated groundwater while working underground in a specific gold mine shaft in the City of Matlosana. The outbreak was perpetuated by at least 3 different but concurrent patterns of transmission: (1) a persistent source of transmission to Zama Zamas due to the presence of acutely ill miners underground and possibly chronic carriers, (2) secondary transmission directly from infected miners to household and community contacts, and (3) transmission to other provinces by infected miners traveling from the City of Matlosana to other provinces. This investigation highlights the value of whole genome sequencing to detect clusters and support epidemiologic investigation of *Salmonella* Typhi outbreaks.

## Notes

***Acknowledgments*.** CED; South African Field Epidemiology Program; North West Environmental Health Office; Communicable Diseases Cluster, City of Matlosana local municipality, Dr Kenneth Kaunda District; North West Provincial Epidemiologist; North West Office of the Premier. We thank all participants of the NICD GERMS-SA Laboratory Surveillance Network for submission of clinical isolates of *Salmonella* species to the NICD; the surveillance network includes laboratories belonging to the Department of Health (National Health Laboratory Services laboratories) and laboratories that form part of the private sector.

***Author contributions***. P. S. conducted the epidemiologic investigations, including patient interviews and data analysis, and created the first draft of this article. L. E. and J. T. conducted epidemiologic investigations. M. N. and B. D. performed serotyping of the isolates to confirm the serovar. A. M. S., N. T., and S. W. conducted the analysis of whole genome sequencing data using core genome multilocus sequence typing. In addition, A. M. S. obtained funding for whole genome sequencing. M. S. and L. J. conducted the epidemiologic investigations including patient interviews. G. M. conducted epidemiologic investigations, including patient interviews and conducting environmental sampling and assessments. W. M. conducted epidemiologic investigations, including patient interviews. G. T. provided oversight role and coordination of the multistakeholder outbreak response activities. All authors participated in reviewing and editing the manuscript.

***Data availability statement*.** Data will be made available upon request.

***Disclaimer.*** The views expressed in this publication are those of the authors and not necessarily those of the UK Department of Health and Social Care or its management agent, Mott MacDonald.

***Patient consent statement***. The authors declare that all activities contributing to this work comply with the ethical standards of the relevant national and institutional committees on human experimentation and with the Helsinki Declaration of 1975, as revised in 2008. NICD outbreak investigations are approved by the research ethics committee at the University of the Witwatersrand, Johannesburg, South Africa (clearance certificate M210752). Verbal informed consent was obtained from cases who were interviewed, and to ensure the protection of personal information, personal identifiers were removed from the data before analysis. Data were kept on a password-encrypted Excel spreadsheet with access limited to the investigators.

***Financial support*.** This work was supported by the SEQAFRICA project (for whole genome sequencing), which is funded by the Department of Health and Social Care’s Fleming Fund using UK aid.

***Potential conflicts of interest***. All authors: No reported conflicts.

## References

[1] Neupane DP, Dulal HP, Song J. Enteric fever diagnosis: current challenges and future directions. Pathogens 2021; 10:410. 33915749 10.3390/pathogens10040410 PMC8065732

[2] Rizzo C, Santantonio M, Coscia MF, Monno R, De Vito D, Rizzo G. Typhoid fever in Italy, 2000–2006. J Infect Dev Ctries 2008; 2:466–8. 19745525 10.3855/jidc.163

[3] Purcell R, Pollard AJ. Elimination of typhoid: possibility or pipe dream? J Paediatr Child Health 2020; 56:1340–2. 32767815 10.1111/jpc.15088

[4] Kanungo S, Dutta S, Sur D. Epidemiology of typhoid and paratyphoid fever in India. J Infect Dev Ctries 2008; 2:454–60. 19745523 10.3855/jidc.161

[5] World Health Organization . Typhoid. Available at: https://www.who.int/health-topics/typhoid#tab=tab_1. Accessed 11 October 2022.

[6] Kumar P, Kumar R. Enteric fever. Indian J Pediatr 2017; 84:227–30. 27796818 10.1007/s12098-016-2246-4

[7] Khanam F, Ross AG, McMillan NAJ, Qadri F. Toward typhoid fever elimination. Int J Infect Dis 2022; 119:41–3. 35338009 10.1016/j.ijid.2022.03.036

[8] GBD 2017 Typhoid and Paratyphoid Collaborators . The global burden of typhoid and paratyphoid fevers: a systematic analysis for the Global Burden of Disease Study 2017. Lancet Infect Dis 2019; 19:369–81. 30792131 10.1016/S1473-3099(18)30685-6 PMC6437314

[9] Garrett DO, Longley AT, Aiemjoy K, et al Incidence of typhoid and paratyphoid fever in Bangladesh, Nepal, and Pakistan: results of the Surveillance for Enteric Fever in Asia Project. Lancet Glob Health 2022; 10:e978–88. 35714648 10.1016/S2214-109X(22)00119-X PMC9210262

[10] Meiring JE, Shakya M, Khanam F, et al Burden of enteric fever at three urban sites in Africa and Asia: a multicentre population-based study. Lancet Glob Health 2021; 9:e1688–96. 34798028 10.1016/S2214-109X(21)00370-3 PMC8609278

[11] Carey ME, MacWright WR, Im J, et al The Surveillance for Enteric Fever in Asia Project (SEAP), Severe Typhoid Fever Surveillance in Africa (SETA), Surveillance of Enteric Fever in India (SEFI), and Strategic Typhoid Alliance Across Africa and Asia (STRATAA) population-based enteric fever studies: a review of methodological similarities and differences. Clin Infect Dis 2020; 71(suppl 2):S102–10. 32725221 10.1093/cid/ciaa367 PMC7388711

[12] Keddy KH, Smith AM, Sooka A, et al The burden of typhoid fever in South Africa: the potential impact of selected interventions. Am J Trop Med Hyg 2018; 99(3 suppl):55–63. 30047360 10.4269/ajtmh.18-0182 PMC6128358

[13] Keddy KH, Sooka A, Ismail H, et al Molecular epidemiological investigation of a typhoid fever outbreak in South Africa, 2005: the relationship to a previous epidemic in 1993. Epidemiol Infect 2011; 139:1239–45. 20875199 10.1017/S0950268810002207

[14] Veltman S, Wilke AR. Groundwater flow mechanisms at Margaret shaft, KOSH gold mining area. In: 2008 International Mine Water Conference Proceedings. Available at: http://www imwa info/docs/imwa_2008/IMWA2008_188_Veltman pdf. Accessed 2012.

[15] STATSSA . City of Matlosana. Available at: https://www.statssa.gov.za/? page_id=993&id=city-of-matlosana-municipality. Accessed 11 October 2022.

[16] Grimont PA, Weill F-X; WHO Collaborating Centre for Reference and Research on Salmonella. Antigenic formulae of the Salmonella serovars. 9th ed. Geneva: World Health Organization, 2007.

[17] Wayne P; Clinical and Laboratory Standards Institute. Performance standards for antimicrobial susceptibility testing: twenty-fourth informational supplement, M100-S28. Berwyn: Clinical and Laboratory Standards Institute, 2018.

[18] Yoshida CE, Kruczkiewicz P, Laing CR, et al The *Salmonella* In Silico Typing Resource (SISTR): an open web-accessible tool for rapidly typing and subtyping draft *Salmonella* genome assemblies. PLoS One 2016; 11:e0147101. 26800248 10.1371/journal.pone.0147101 PMC4723315

[19] Wong VK, Baker S, Connor TR, et al An extended genotyping framework for *Salmonella enterica* serovar Typhi, the cause of human typhoid. Nat Commun 2016; 7:12827. 27703135 10.1038/ncomms12827 PMC5059462

[20] Pulles W, Banister S, Van Biljon M. The development of appropriate procedures towards and after closure of underground gold mines from a water management perspective: report 1215/1. Pretoria: Water Research Commission; **2005**.

[21] McAteer J, Derado G, Hughes M, et al Typhoid fever in the US pediatric population, 1999–2015: opportunities for improvement. Clin Infect Dis 2021; 73:e4581–9. 33247585 10.1093/cid/ciaa914

[22] Fatima M, Kumar S, Hussain M, et al Morbidity and mortality associated with typhoid fever among hospitalized patients in Hyderabad district, Pakistan, 2017–2018: retrospective record review. JMIR Public Health Surveill 2021; 7:e27268. 33999000 10.2196/27268 PMC8167610

[23] Makungo UB, Ramutshila TE, Mabotja MC, et al Epidemiological investigation of a typhoid fever outbreak in Sekhukhune district, Limpopo province, South Africa—2017. S Afr J Infect Dis 2020; 35:107. 34485467 10.4102/sajid.v35i1.107 PMC8378196

[24] Olsen SJ, Bleasdale SC, Magnano AR, et al Outbreaks of typhoid fever in the United States, 1960–99. Epidemiol Infect 2003; 130:13–21. 12613741 10.1017/s0950268802007598 PMC2869934

[25] Khanam F, Darton TC, Meiring JE, et al *Salmonella* Typhi stool shedding by patients with enteric fever and asymptomatic chronic carriers in an endemic urban setting. J Infect Dis 2021; 224(12 suppl 2):S759–63. 34586391 10.1093/infdis/jiab476 PMC8687075

[26] Sohn S, Xercavins M, Llovet T, et al Epidemiology of an unusually prolonged outbreak of typhoid fever in Terrassa, Spain. Clin Infect Dis 1997; 24:506–10. 9114207 10.1093/clinids/24.3.506

[27] Hancock-Allen J, Cronquist AB, Peden J, Adamson D, Corral N, Brown K. Notes from the field: typhoid fever outbreak associated with an asymptomatic carrier at a restaurant—Weld County, Colorado, 2015. MMWR Morb Mortal Wkly Rep 2016; 65:606–7. 27310090 10.15585/mmwr.mm6523a4

[28] Scott NS, Paterson JM, Seale H, Truman G. Chronic carriage and familial transmission of typhoid in western Sydney. Commun Dis Intell Q Rep 2014; 38:E24–5. 25409351 10.33321/cdi.2014.38.6

[29] Kobayashi T, Kutsuna S, Hayakawa K, et al Case report: an outbreak of food-borne typhoid fever due to *Salmonella enterica* serotype Typhi in Japan reported for the first time in 16 years. Am J Trop Med Hyg 2016; 94:289–91. 26621565 10.4269/ajtmh.15-0484 PMC4751929

[30] Egoz N, Shihab S, Leitner L, Lucian M. An outbreak of typhoid fever due to contamination of the municipal water supply in northern Israel. Isr J Med Sci 1988; 24:640–3. 3215755

[31] Meyer Sauteur PM, Stevens MJA, Paioni P, et al Siblings with typhoid fever: an investigation of intrafamilial transmission, clonality, and antibiotic susceptibility. Travel Med Infect Dis 2020; 34:101498. 31580900 10.1016/j.tmaid.2019.101498

[32] National Institute for Communicable Diseases . Communicable disease communique. Johannesburg: National Institute for Communicable Diseases, 2002.

[33] Opare J, Ohuabunwo C, Afari E, et al Outbreak of cholera in the East Akim Municipality of Ghana following unhygienic practices by small-scale gold miners, November 2010. Ghana Med J 2012; 46:116. 23661823 PMC3645159

[34] Diaz-Guevara P, Montaño LA, Duarte C, et al Surveillance of *Salmonella enterica* serovar Typhi in Colombia, 2012–2015. PLoS Negl Trop Dis 2020; 14:e0008040. 32155148 10.1371/journal.pntd.0008040 PMC7083327

[35] Loharikar A, Newton A, Rowley P, et al Typhoid fever outbreak associated with frozen mamey pulp imported from Guatemala to the western United States, 2010. Clin Infect Dis 2012; 55:61–6. 22423132 10.1093/cid/cis296

[36] Bano-Zaidi M, Aguayo-Romero M, Campos FD, et al Typhoid fever outbreak with severe complications in Yucatan, Mexico. Lancet Glob Health 2018; 6:e1062–3. 30223978 10.1016/S2214-109X(18)30312-7

[37] Cruz Espinoza LM, McCreedy E, Holm M, et al Occurrence of typhoid fever complications and their relation to duration of illness preceding hospitalization: a systematic literature review and meta-analysis. Clin Infect Dis 2019; 69(suppl 6):S435–48. 31665781 10.1093/cid/ciz477 PMC6821330

[38] Marchello CS, Birkhold M, Crump JA. Complications and mortality of typhoid fever: a global systematic review and meta-analysis. J Infect 2020; 81:902–10. 33144193 10.1016/j.jinf.2020.10.030 PMC7754788

[39] Bulage L, Masiira B, Ario AR, et al Modifiable risk factors for typhoid intestinal perforations during a large outbreak of typhoid fever, Kampala Uganda, 2015. BMC Infect Dis 2017; 17:641. 28946853 10.1186/s12879-017-2720-2 PMC5613338

[40] Madimu T . Illegal gold mining and the everyday in post-apartheid South Africa. Rev Afr Political Econ 2022; 49:436–51.

